# Masitinib as an adjunct therapy for mild-to-moderate Alzheimer's disease: a randomised, placebo-controlled phase 2 trial

**DOI:** 10.1186/alzrt75

**Published:** 2011-04-19

**Authors:** François Piette, Joël Belmin, Hélène Vincent, Nicolas Schmidt, Sylvie Pariel, Marc Verny, Caroline Marquis, Jean Mely, Laurence Hugonot-Diener, Jean-Pierre Kinet, Patrice Dubreuil, Alain Moussy, Olivier Hermine

**Affiliations:** 1 Hôpital Charles Foix, Service de Médecine, Bâtiment Louis Ramond, 7 avenue de la République, 94205 Ivry-Sur-Seine, France; 2 Hôpital Charles Foix, Service de Gériatrie et Consultation Mémoire, Hôpital Charles Foix, 7 avenue de la République, 94200 Ivry-sur-Seine, France; 3 Université UPMC-Paris 6, 4 place Jussieu, 75005 Paris, France; 4 Centre d'investigation libéral des troubles de mémoire, 6 av Alsace-Lorraine, 92500 Rueil Malmaison, France; 5 Hôpital Pitié-Salpêtrière et Université UPMC-Paris 6, Centre de Gériatrie, 47-83 bd de l'Hôpital, 75651 Paris, France; 6 Centre Hospitalier de la Région de St Omer, Neurologie et Gériatrie, Route des Blendecques, 62505 Helfaut, France; 7 Medforma, 108 bis Bd A. Blanqui, 75013 Paris, France; 8 Laboratory of Allergy and Immunology, Department of Pathology, Beth Israel Deaconess Medical Center and Harvard Medical School, 330 Brookline Avenue, Boston, MA 02215, USA; 9 Inserm U891, Centre de Recherche en Cancérologie de Marseille, Signalisation, Hématopoïèse et Mécanismes de l'Oncogenèse, Centre de Référence des Mastocytoses, 13009 Marseille, France; 10 Institut Paoli-Calmettes, 13009 Marseille, France; 11 Université de la Méditerranée, 27 Bd Leï roure, 13009 Marseille, France; 12 AB Science, 3 avenue George V, 75008 Paris, France; 13 Service d'hématologie adulte, centre de référence sur la mastocytose, CNRS UMR 8147, Hôpital Necker Assistance publique hôpitaux de Paris, Université Paris V - René Descartes, 149 rue de Sèvres, 75015 Paris, France

## Abstract

**Introduction:**

Neuroinflammation is thought to be important in Alzheimer's disease pathogenesis. Mast cells are a key component of the inflammatory network and participate in the regulation of the blood-brain barrier's permeability. Masitinib, a selective oral tyrosine kinase inhibitor, effectively inhibits the survival, migration and activity of mast cells. As the brain is rich in mast cells, the therapeutic potential of masitinib as an adjunct therapy to standard care was investigated.

**Methods:**

A randomised, placebo-controlled, phase 2 study was performed in patients with mild-to-moderate Alzheimer's disease, receiving masitinib as an adjunct to cholinesterase inhibitor and/or memantine. Patients were randomly assigned to receive masitinib (*n *= 26) (starting dose of 3 or 6 mg/kg/day) or placebo (*n *= 8), administered twice daily for 24 weeks. The primary endpoint was change from baseline in the Alzheimer's Disease Assessment Scale - cognitive subscale (ADAS-Cog) to assess cognitive function and the related patient response rate.

**Results:**

The rate of clinically relevant cognitive decline according to the ADAS-Cog response (increase >4 points) after 12 and 24 weeks was significantly lower with masitinib adjunctive treatment compared with placebo (6% vs. 50% for both time points; *P *= 0.040 and *P *= 0.046, respectively). Moreover, whilst the placebo treatment arm showed worsening mean ADAS-Cog, Alzheimer's Disease Cooperative Study Activities of Daily Living Inventory, and Mini-Mental State Examination scores, the masitinib treatment arm reported improvements, with statistical significance between treatment arms at week 12 and/or week 24 (respectively, *P *= 0.016 and 0.030; *P *= 0.035 and 0.128; and *P *= 0.047 and 0.031). The mean treatment effect according to change in ADAS-Cog score relative to baseline at weeks 12 and 24 was 6.8 and 7.6, respectively. Adverse events occurred more frequently with masitinib treatment (65% vs. 38% of patients); however, the majority of events were of mild or moderate intensity and transitory. Severe adverse events occurred at a similar frequency in the masitinib and placebo arms (15% vs. 13% of patients, respectively). Masitinib-associated events included gastrointestinal disorders, oedema, and rash.

**Conclusions:**

Masitinib administered as add-on therapy to standard care during 24 weeks was associated with slower cognitive decline in Alzheimer's disease, with an acceptable tolerance profile. Masitinib may therefore represent an innovative avenue of treatment in Alzheimer's disease. This trial provides evidence that may support a larger placebo-controlled investigation.

**Trial registration:**

Clinicaltrials.gov NCT00976118

## Introduction

Alzheimer's disease (AD) is a degenerative neurological disorder and the most common cause of dementia and disability in the older patient [1]. With no known cure and the currently available treatments only able to temporarily ease symptoms, additional therapeutic options are required. New therapeutic approaches include minimising accumulation of amyloid-beta (Aβ) peptides in the brain [2,3] or targeting cells and signalling pathways implicated in neuronal destruction associated with neuroinflammation [4-6].

Mast cells, which are found on both sides of the blood-brain barrier (BBB) [7-9], release large amounts of proinflammatory mediators and therefore play a prominent role in sustaining the inflammatory network of the central nervous system [10]. Moreover, their ability to regulate the BBB's permeability may also be of therapeutic significance; a defective BBB being a common finding in neuroinflammatory and neurodegenerative diseases, including AD [8,11-14]. Masitinib mesilate, the investigatory drug of the present study, is a selective tyrosine kinase inhibitor that targets c-Kit, platelet-derived growth factor receptors (PDGFR), and, to a lesser extent, Lyn, Fyn, and the FAK pathway, without inhibiting kinases of known toxicities [15]. By combined targeting of c-Kit and Lyn, masitinib is particularly efficient in controlling the survival, differentiation, and degranulation of mast cells, and thus indirectly controlling the array of proinflammatory and vasoactive mediators the cells can release. Indeed, promising results have been reported from human clinical trials of masitinib in inflammatory diseases such as rheumatoid arthritis and asthma [16,17].

To investigate the hypothesis that masitinib's targeted inhibitory action on mast cells may reduce the symptoms of AD, its efficacy and safety was assessed as compared with a placebo. Masitinib was administered orally as an adjunct therapy to standard care in patients with mild-to-moderate AD.

## Materials and methods

### Study design and treatment

A multicentre (12 study centres across France), double-blind, randomised, placebo-controlled, parallel-group study of oral masitinib as add-on therapy in mild-to-moderate AD patients, treated over 24 weeks, was performed. Patients were treated concomitantly with a stable dose of anti-cholinesterase (donepezil, rivastigmine, or galantamine) and/or memantine throughout the study. To evaluate the optimal starting dose for masitinib in AD, dose ranging was performed using masitinib groups of 3 or 6 mg/kg/day. Patients were randomly allocated to the two masitinib initial dose groups and placebo group in a 5:5:3 ratio. A centralised randomisation schedule for packaging and labelling was generated and held by a third-party service (Cardinal Systems, Paris, France), and was implemented using an interactive voice response system. All participants and study personnel were blinded to treatment allocated over the study's duration. For each patient, all efficacy and safety parameters were recorded on the first day of treatment (baseline), with patient visits thereafter scheduled for weeks 2, 4, 8, 12, and 24. Haematology and biochemistry analyses were performed regularly over the study period.

Masitinib was provided by AB Science (Paris, France) in 100 or 200 mg nondivisible coated tablets, to be administered orally twice daily. For a patient weighing 66 kg to receive the target dose of 6 mg/kg/day, a total of 396 mg was therefore required, administered as two 200 mg tablets. Composition and dispensing of the masitinib and placebo treatments were identical except for the amount of active ingredient contained. Blinded dose adjustments of 1.5 mg/kg/day were permitted according to efficacy and safety outcome, with the dosage being incremented in cases of insufficient response accompanied by minimal toxicity at weeks 4 and 8 to a maximum dose of 7.5 mg/kg/day (that is, one additional 100 mg tablet is required for a 66 kg patient previously receiving 6 mg/kg/day to achieve the theoretical dose of 495 mg). Following predetermined criteria, treatment could be temporarily interrupted and/or the dosage decreased by 1.5 mg/kg/day in the event of toxicity.

The present investigation was carried out in accordance with the Declaration of Helsinki and approved by the national health authorities and a local central ethics committee (Comité de Protection des Personnes Ile-de-France II).

### Eligibility criteria

Patients aged ≥50 years diagnosed with mild-to-moderate AD (according to Diagnostic and Statistical Manual of Mental Disorders IV criteria, and to National Institute of Neurological and Communicative Disorders and Stroke-Alzheimer's Disease and Related Disorders Association criteria), with a baseline Mini-Mental State Examination (MMSE) score between 12 and 26 and a baseline Clinical Dementia Rating (CDR) of 1 or 2, were eligible to participate in the present study. Patients must have been treated for a minimum of 6 months with stable doses of cholinesterase inhibitors (donepezil, rivastigmine, or galantamine), and/or memantine for a minimum of 3 months at study entry, with no dose change foreseen during the study. The presence of a reliable caregiver was required, with both the patient and the caregiver providing written informed consent.

Patients with severe AD or any other cause of dementia were excluded, as were those receiving cognitive enhancers or disease modifiers other than donepezil, galantamine, rivastigmine, or memantine. The following conditions were exclusion criteria: delusions or delirium, uncontrolled depression, evidence of psychosis and/or use of antipsychotic drugs, a history of significant psychotic/psychiatric disorders, active infection, treatment with an investigational agent within 4 weeks of inclusion, or a history of poor compliance.

### Efficacy and safety assessment

The primary endpoint was the Alzheimer's Disease Assessment Scale - cognitive subscale (ADAS-Cog) to assess cognitive function. Response was expressed as the mean difference in ADAS-Cog at week 24 relative to baseline, and as the proportion of patients achieving *a priori *response thresholds at week 24 (defined by a blinded Data Review Committee prior to unblinding). Improvement was defined as a decrease ≥4 in ADAS-Cog score, worsening as an increase ≥4, and any other change was considered as stable. Secondary endpoints included: the Alzheimer's Disease Cooperative Study Activities of Daily Living Inventory (ADCS-ADL) to assess self-care; the Clinician's Interview-Based Impression of Change-plus caregiver input (CIBIC-Plus) to assess overall clinical response; the MMSE to evaluate cognitive function; and the CDR to characterise cognitive and functional performance.

Safety was assessed throughout the study via physical examinations, vital signs, clinical laboratory evaluations and monitoring of adverse events (AEs), with all AEs recorded regardless of causality.

### Statistical analysis

Efficacy analyses were performed on the intent-to-treat and per-protocol populations. The intent-to-treat population was defined as all randomised patients, and the per-protocol population was defined as a subgroup of the intent-to-treat population that presented no major protocol deviations. Analysis was conducted using three possible datasets: (i) imputation of missing values according to the last observation carried forward (LOCF) methodology; (ii) an observed cases methodology (that is, the absence of data imputation); and (iii) considering patients with missing data as nonresponders. Due to circumstances not directly related to the study (an investigator died), it was not possible to collect week 24 measurements of patients from one study centre (*n *= 8; consisting of seven patients from the masitinib group and one patient from the placebo group). Week 12 data for this centre were therefore imputed for week 24 in the observed cases analysis. Descriptive statistics were used to analyse the safety population (all patients receiving at least one drug administration). Quantitative variables were compared using a nonparametric Wilcoxon rank sum test, and the Fisher's exact test was used for comparing categorical variables. The Cochran-Mantel-Haenzel test was also used for ordinal variables.

## Results

### Participant flow

**Figure 1:**
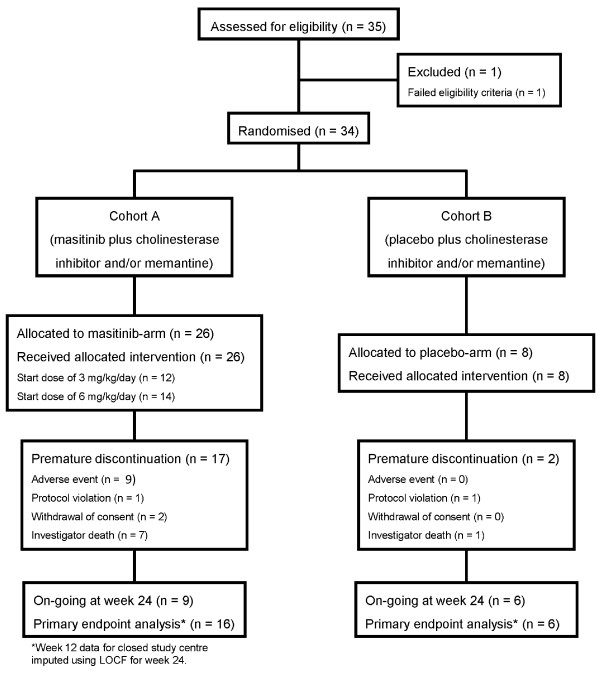
**Consort diagram**. LOCF, last observation carried forward.

A total of 35 patients were screened between February 2006 and August 2008, of which 34 were randomised: 26 patients into the masitinib group (*n *= 12 and *n *= 14 at 3 and 6 mg/kg/day, respectively) and eight patients into the placebo group (Figure 1). Overall, patient baseline characteristics were well balanced between treatment arms (Table 1), although the placebo group had a comparatively higher mean age (78 years vs. 72 years; *P *= 0.167) and ADAS-Cog score (25.6 vs. 18.8; *P *= 0.161). No protocol deviations were reported as a result of poor test treatment compliance.

**Table 1:** Summary of baseline characteristics

Characteristic	Masitinib treatment (*n *= 26)	Placebo (*n *= 8)	*P *value
Age (years)	72 ± 12	78 ± 11	0.167
Gender (male/female)	11 (42%)/15 (58%)	2 (25%)/6 (75%)	0.444
Time since diagnosis (years)	1.7 ± 1.1	1.8 ± 0.8	0.320
ADAS-Cog (0 to 70)	18.8 ± 6.7	25.6 ± 12.1	0.161
MMSE (0 to 30)	19.1 ± 3.9	18.0 ± 4.4	0.650
CDR (1/2)	21 (81%)/5 (19%)	6 (75%)/2 (25%)	1.000
ADCS-ADL (0 to 70)	47.1 ± 11.2	45.9 ± 18.0	0.850
Concomitant Alzheimer's treatment			
Cholinesterase inhibitors	26 (100%)	8 (100%)	0.180
Memantine	4 (15%)	2 (25%)	0.610

Data presented as mean ± standard deviation or number (%). ADAS-Cog, Alzheimer's Disease Assessment Scale - cognitive subscale; ADCS-ADL, Alzheimer's Disease Cooperative Study Activities of Daily Living; CDR, Clinical Dementia Rating; MMSE, Mini-Mental State Examination.

As required by the inclusion criteria, all patients were receiving a stable dose of a cholinesterase inhibitor, with eight patients receiving concomitant cholinesterase inhibitors and memantine. Patients were also required to maintain a stable dose of these drugs during the course of the study; however, one patient from the masitinib group discontinued cholinesterase inhibitor treatment (donepezil) on the first day of the study and was withdrawn on day 29 due to this major protocol deviation. Minor concomitant treatment protocol deviations were noted for two patients who did not maintain a stable dose of cholinesterase inhibitor and/or memantine on study. One patient in the masitinib group changed type of medication during the extension phase (donepezil 10 mg modified to memantine 10 mg), and one patient from the placebo group changed dose of donepezil from 10 to 5 mg after 8 weeks of treatment; both of these patients, however, were retained for analyses. The mean actual masitinib dose received was 4.1 ± 1.3 and 6.2 ± 0.6 mg/kg/day in the theoretical 3 and 6 mg/kg/day groups, respectively, reflecting that dose increments occurred more frequently in the initial 3 mg/kg/day group.

In total, 19/34 patients (56%) withdrew before the planned completion of treatment; 17/26 patients (65%) from the masitinib group and 2/8 patients (25%) from the placebo group. If the 8/34 patients (24%) who were withdrawn due to closure of their treatment centre for circumstances unrelated to the study are disregarded, then the associated patient withdrawal rates become 10/26 patients (38%) from the masitinib group and 1/8 patient (12.5%) from the placebo group. Premature withdrawal instigated by the investigator on grounds of treatment-related AEs was reported for 7/26 masitinib-treated patients (27%) compared with no patients in the placebo group.

### Efficacy

Unless stated otherwise, data from the intent-to-treat population according to the observed cases dataset analysis (with LOCF data imputation at week 24 for those patients withdrawn due to closure of their centre at week 12) are presented hereafter. A summary of efficacy data at weeks 12 and 24 is presented in Table 2.

**Table 2:** Summary of efficacy outcomes at weeks 12 and 24

	Week 12			Week 24		
Treatment arm	Masitinib treatment	Placebo	*P *value	Masitinib treatment	Placebo	*P *value
ADAS-Cog^a^						
Evaluable patients	17	6		16	6	
Improvement^b^	7 (41%)	1 (17%)	0.369	6 (38%)	1 (17%)	0.616
Worsening^b^	1 (6%)	3 (50%)	0.040	1 (6%)	3 (50%)	0.046
Mean absolute change	-2.6 ± 3.6	4.2 ± 6.6	0.016	-1.8 ± 6.1	5.8 ± 7.9	0.030
ADCS-ADL^c^						
Evaluable patients	16	6		15	6	
Improvement^d^	8 (50%)	0 (0%)	0.051	9 (60%)	1 (17%)	0.149
Worsening^d^	5 (31%)	3 (50%)	0.624	4 (27%)	3 (50%)	0.354
Mean absolute change	6.9 ± 10.9	-4.2 ± 6.9	0.035	5.5 ± 15.8	-1.8 ± 7.0	0.128
MMSE^c^						
Evaluable patients	17	7		16	7	
Mean absolute change	0.1 ± 2.5	-2.1 ± 2.5	0.047	-0.1 ± 4.3	-3.3 ± 3.3	0.031
CDR response^e^						
Evaluable patients	17	7	0.778*	16	7	0.293*
Response	2 (12%)	1 (14%)		3 (19%)	1 (14%)	
No change	14 (82%)	5 (71%)		12 (75%)	4 (57%)	
Worsening	1 (6%)	1 (14%)		1 (6%)	2 (29%)	
CIBIC-Plus						
Evaluable patients	17	6	0.292*	16	6	0.474*
Response (1 to 3)	1 (6%)	1 (17%)		2 (13%)	0	
No change (4)	14 (82%)	2 (33%)		12 (75%)	5 (83%)	
Worsening (5 to 7)	2 (12%)	3 (50%)		2 (13%)	1 (17%)	

Summary of efficacy outcomes at weeks 12 and 24 according to observed cases dataset analysis on the intent-to-treat population. Data presented as mean ± standard deviation, or number (%). Week 12 data for closed study centre (*n *= 8 patients) was imputed using last observation carried forward for week 24. ADAS-Cog, Alzheimer's Disease Assessment Scale - cognitive subscale; ADCS-ADL, Alzheimer's Disease Cooperative Study Activities of Daily Living; CDR, Clinical Dementia Rating; CIBIC-plus, Clinician's Interview-Based Impression of Change-plus caregiver input; MMSE, Mini-Mental State Examination. ^a^Negative change reflects improvement. ^b^ADAS-Cog response criteria were improvement (decrease ≥4), worsening (increase ≥4). ^c^Positive change reflects improvement. ^d^ADCS-ADL response criteria were improvement (increase ≥3), worsening (decrease <0). ^e^CDR response criteria were positive response (decrease > 0), worsening (increase > 0). *Global *P *value.

**Figure 2:**
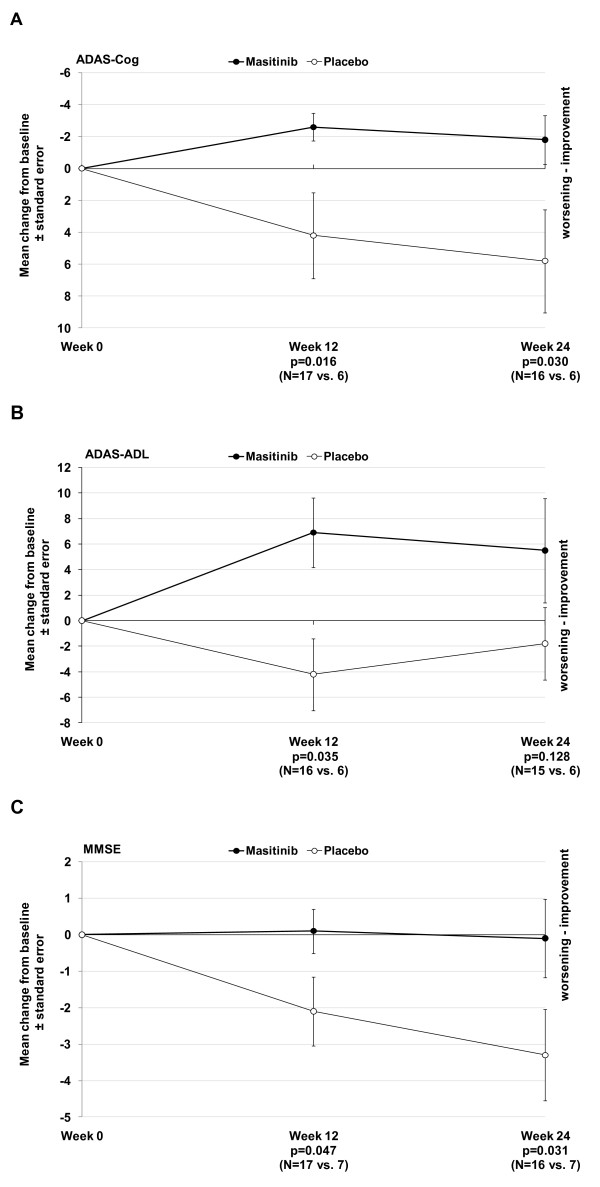
**Summary of efficacy data at weeks 12 and 24**. Mean change from baseline to week 24 in **(a) **Alzheimer's Disease Assessment Scale - cognitive subscale (ADAS-Cog), **(b) **Alzheimer's Disease Cooperative Study Activities of Daily Living Inventory (ADCS-ADL) and **(c) **Mini-Mental State Examination (MMSE), according to observed cases dataset analysis on the intent-to-treat population. *N*, number of evaluable patients at each time point (masitinib-treated versus placebo, respectively).

Decline of cognitive function, as assessed by the primary endpoint of ADAS-Cog responder rate, was significantly higher in the placebo arm compared with the masitinib treatment arm after 12 and 24 weeks (50% vs. 6% for both; *P *= 0.040 and *P *= 0.046, respectively). Change in ADAS-Cog score relative to baseline showed a significant difference between the masitinib and placebo groups at week 12 (*P *= 0.016), which was maintained at week 24 (*P *= 0.030) (Table 2 and Figure 2a). The mean treatment effect was 6.8 and 7.6, respectively. At both time points an increase (that is, decline in function) was observed in the placebo arm's ADAS-Cog mean scores, whereas the masitinib treatment arm registered mean decreases in scores (that is, improvement in function).

The proportion of responders showing an improvement in daily living activities, defined as an ADCS-ADL increase ≥3, was higher in the masitinib treatment arm compared with the placebo arm at weeks 12 and 24; respectively, 50% versus 0% (*P *= 0.051) and 60% versus 16.7%, (*P *= 0.149) (Table 2). The mean change in ADCS-ADL relative to baseline showed significant improvement for the masitinib treatment arm compared with the placebo arm at week 12 (*P *= 0.035), although this improvement was no longer statistically significant at week 24 (*P *= 0.128) (Figure 2b). At both time points a mean increase (that is, improvement in function) was observed for masitinib treatment, while a mean decrease (that is, decline in function) was observed for placebo administration.

Assessment of the MMSE score revealed a significant difference between groups after 12 weeks (*P *= 0.047) and 24 weeks of treatment (*P *= 0.031) (Figure 2c); masitinib-treated patients having steady MMSE scores relative to baseline compared with negative absolute changes in the placebo group, representing stability or decline in cognitive function, respectively. The CIBIC-plus evaluation showed a worsening score for a lower proportion of patients in the masitinib treatment arm compared with the placebo arm at week 12: 2/17 patients (12%) versus 3/6 patients (50%), respectively (*P *= 0.089). This difference was no longer apparent at week 24; however, 2/16 patients (12.5%) did register an improved response following masitinib treatment compared with none in the placebo group. CDR response analysis at 24 weeks showed 15/16 (94%) masitinib-treated patients remained stable or improved relative to baseline as compared with 5/7 patients (71%) receiving placebo. Likewise, more patients showed deterioration under placebo compared with masitinib treatment; however, no significant differences between treatment arms were reported (Table 2).

Parallel masitinib-treatment groups at different initial dose levels were studied to determine the optimal starting dose of masitinib, with dose adjustments possible in cases of insufficient response. Dose augmentation occurred in 54% versus 7% of patients in the 3 and 6 mg/kg/day groups, respectively. Furthermore, a higher rate of cognitive improvement according to decrease in ADAS-Cog score ≥4 points was observed in the 6 mg/kg/day masitinib subpopulation (31% vs. 17% at 3 mg/kg/day). These data suggest that a masitinib starting dose of 6 mg/kg/day is optimal for future investigations.

### Safety

Frequent AEs (with an incidence ≥5%) or any severe event reported over the 24-week study are presented in Table 3. Overall, AEs were more common in the masitinib group compared with the placebo group (17/26 patients (65%) versus 3/8 patients (38%), respectively), with the most frequent toxicities being oedema irrespective of localisation (31%, including 19% of patients with peripheral oedema and 15% of patients with eyelid oedema), gastrointestinal (diarrhoea 23%, nausea 15%, vomiting 12%), rash (19%), and metabolic or general disorders. The majority of masitinib-associated AEs were of mild-to-moderate intensity and were transitory. Severe AEs occurred at a similar frequency in the masitinib treatment and placebo arms (4/26 patients (15%) and 1/8 patient (13%), respectively) - the masitinib group reporting occurrences of rash, anorexia, nausea, asthenia, and transaminase increases (with concomitant mild neutropaenia and leukopaenia). A total of seven patients reported at least one nonfatal serious AE, consisting of 1/8 patient (12.5%) from the placebo group and 6/26 patients (23%) from the masitinib group. Of the latter, 5/26 patients (19%) were suspected to be treatment related, with a maximum reaction intensity of severe, moderate or mild being reported for two patients, two patients, and one patient, respectively. No deaths occurred during this study. Seven masitinib patients (27%) experienced treatment-related AEs that resulted in treatment discontinuation, including all four patients with severe AEs and three of the five patients with nonfatal serious AEs. Comparison of safety between the masitinib 3 and 6 mg/kg/day groups showed a similar overall frequency of AEs (69% vs. 62%, respectively); although there was a slightly elevated occurrence of severe AEs reported in the 6 mg/kg/day group, three patients (23%) compared with just one patient (8%) in the 3 mg/kg/day group.

**Table 3:** Number of patients with at least one adverse event (> 5%), according to intensity

	Masitinib treatment (*n *= 26)		Placebo (*n *= 8)	
	All	Severe	All	Severe
At least one adverse event	17 (65%)	4 (15%)	3 (38%)	1 (13%)
Oedema - all	8 (31%)			
Diarrhoea	6 (23%)			
Rash - all	5 (19%)	2 (8%)		
Anorexia	4 (15%)	2 (8%)		
Nausea	4 (15%)	1 (4%)		
Vomiting	3 (12%)			
Asthenia	3 (12%)	1 (4%)		
Bronchitis	2 (8%)			
Weight decreased	2 (8%)		1 (13%)	
Transaminases increased	2 (8%)	1 (4%)		
Arthralgia	2 (8%)			
Depression	2 (8%)		1 (13%)	
Balance disorder			1 (13%)	1 (13%)

## Discussion

Within the limitations inherent to such relatively small phase 2 studies, these results suggest that oral masitinib may have benefits in patients with mild-to-moderate AD. The mechanisms underlying this response remain to be elucidated; as orally administered masitinib is unlikely to have effectively penetrated the BBB, however, we may assume its mechanism of action must be indirect, originating outside the BBB [18]. A growing body of evidence implicates Aβ peptides (predominantly Aβ42) as being the main mediator of neurotoxicity in AD [2,3]. Additionally, neuroinflammation is thought to be a major contributor in the pathogenesis of AD [4-6]. Therapies are therefore being sought that reduce Aβ-peptide accumulation and inflammatory response in the brain [19]. Moreover, it has been proposed that blood-borne Aβ peptides could represent a substantial and chronic source of soluble, exogenous Aβ peptides [11]. Although plasma levels of Aβ peptides are about 20-fold lower than cerebrospinal fluid levels, altered BBB function could provide a route for blood-borne Aβ peptides to contribute to AD. The brain is usually protected from this reservoir of Aβ peptides by the BBB; however, there is evidence suggesting that the BBB is defective in AD patients [11-14], conceivably allowing an influx of exogenous Aβ peptides and other blood-borne compounds. Therapies to maintain or reinforce the integrity of the BBB could thus be beneficial in AD.

The possible contribution of mast cells in the physiopathology of AD remains a relatively unknown factor [7,20]. Mast cells reside within the brain, where they are constitutively active or can be activated by a wide range of stimuli, including Aβ peptides [20]. It has been shown that mast cells are able to cross the BBB and their numbers may rapidly increase in response to physiological manipulations [7-9]. Because mast cells release large amounts of proinflammatory mediators, they play a prominent role in sustaining the inflammatory network [10]. Additionally, perivascular localised mast cells secrete numerous vasoactive molecules that regulate BBB permeability [21,22]. Masitinib is an effective targeted therapy against mast cells, exerting a direct proapoptotic, anti-migratory, and anti-activation action [15]. We therefore propose that the positive response observed from orally administered masitinib is due in part to its inhibitory action of mast cells. In one possible scenario, inhibition of mast cell mediators and apoptosis of mast cells localised at the BBB would effectively reduce BBB permeability, thereby reinforcing its integrity and stemming the accumulation of exogenous Aβ peptides in the brain with a subsequent decrease in plaque formation, inflammatory response and possibly tau hyperphosphorylation (according to the amyloid hypothesis). Additionally, the influx of proinflammatory molecules released from peripheral mast cells would be reduced, as well as Aβ-induced activation of brain mast cells, further decreasing neuroinflammation and migration of mast cells to the brain. Inhibition of mast cells peripheral to the BBB could therefore impact on the main pathological features of AD.

In the event that masitinib could pass through the BBB and accumulate to a sufficiently high therapeutic concentration - for example, via inflammation-induced permeability or compromised BBB - then several direct mechanisms of action are possible. Neuroinflammation could be reduced through direct inhibition of brain mast cells and modulation of microglial activity via disruption of the SCF/c-Kit signalling pathway [15,23]. Damage caused by neurofibrillary tangles or Aβ protein could be reduced via masitinib's targeting of Fyn or the FAK pathway, kinases that have been implicated in the phosphorylation pathway of Tau protein and Aβ-induced cognitive impairment [24-26]. It has also been shown that activation of PDGFR, Src, and Rac1 could be relevant for the generation of Aβ by neurons, and that new targets for therapeutic interventions could be found in this pathway [27]; masitinib's inhibition of PDGFR might therefore possibly inhibit Aβ generation through disruption of this pathway. These mechanisms are only applicable, however, if masitinib crosses into the brain in sufficient concentration, which was not assessed in the present study.

The current study has shown that masitinib administered as an adjunct to standard treatments during 24 weeks may possibly slow the rate of cognitive decline of AD compared with placebo, as evident from the sustained and statistically significant response in ADAS-Cog. Significant improvement in cognitive function and functional capacity compared with placebo was also evident through the mean change in ADAS-Cog, MMSE, and ADCS-ADL values relative to baseline - findings additionally supported by favourable response in the CIBIC-plus and CDR analyses. Such broad benefits are desirable in AD, effectively translating into an improved quality of life. One should note, however, during the 24-week study period that those patients treated with placebo in association with cholinesterase inhibitors and/or memantine experienced an unusually high rate of decline in their status when compared with reported studies [28,29]. This observation may reflect a bias related to the higher age and baseline ADAS-Cog score of the placebo group, with the possible implication that this group would experience a faster cognitive decline resulting in an overestimation of treatment effect. For progressive diseases such as AD, the use of LOCF analysis is inclined to underestimate cognitive decline; however, due to the relatively high patient attrition rate, exasperated by the closure of one centre, it was considered appropriate to retain the patients from this closed centre via LOCF analysis for the week 24 analysis. This again may have tended to overestimation of the treatment effect at week 24, although it should be emphasised that significant treatment response was observed at week 12 for which not data imputation was performed.

Because it is likely that these factors will have resulted in some degree of overestimation to the observed response, a number of complementary analyses were performed. In general, results of the presented ADAS-Cog analysis were supported by alternative sensitivity analyses (see Table S1 in Additional file 1). For example, in the observed cases dataset (without data imputation for the closed study centre), the mean treatment effect at week 24 was similar at 7.2 (compared with 7.6), although the change in ADAS-Cog score relative to baseline no longer reached statistical significance (*P *= 0.182) between treatment groups. A higher decline of cognitive function, as assessed by the ADAS-Cog responder rate, was also recorded in the placebo arm compared with the masitinib treatment arm at week 24 (60% vs. 11%, respectively; *P *= 0.095). Considering analysis of the intent-to-treat population by last observation carried forward, the recorded mean treatment effect was less pronounced at week 24 (3.3 at week 12 and 4.0 at week 24); however, an increase (that is, decline in function) was observed in the placebo arm's ADAS-Cog mean scores at both time points, whereas the masitinib treatment arm registered mean decreases (that is, improvement in function). Additionally, to investigate the impact of treatment groups not being comparable at baseline, a multivariate logistic model was constructed (with adjustment on sex, age and ADAS-Cog score at baseline) to test the effect of masitinib on worsening ADAS-Cog score. This model showed that the parameters sex (*P *= 0.754) and ADAS-Cog score (*P *= 0.974) had no particular effect, while age (*P *= 0.232) showed a nonsignificant effect. Overall, a positive - albeit nonsignificant - treatment response was still observed (*P *= 0.247). Taken together, these complementary analyses suggest the positive treatment response observed is unlikely to be entirely due to baseline or patient withdrawal effects.

While the safety profile in the present study population showed a higher rate of toxicity (approximately 1.7-fold increase) with masitinib as an adjunct therapy compared with standard (placebo) therapy, the majority of AEs reported were mild to moderate and transient, with few severe side effects. The most frequent masitinib-associated AEs were consistent with the known safety profile of tyrosine kinase inhibitors - notably oedema, rash, nausea, vomiting, and diarrhoea, which are generally considered manageable with symptomatic treatments. A comparison of masitinib's safety profile in this study with that of other masitinib phase 2 nononcology studies shows comparable results, indicating that treatment of this older population with masitinib (median age 75.5 years vs. 49 years for all other studies) remains manageable with no indication of additive toxicity when used in combination with cholinesterase inhibitor and/or memantine (Table 4). This comparison of masitinib-related AEs according to population age also revealed that although the current study population experienced lower rates of overall and severe AEs compared with the pooled masitinib population, it had an equivalent rate of AE-related patient premature withdrawal. This discrepancy may reflect an understandably cautious approach to AEs given that this was an older population. The common misperception that tyrosine kinase inhibitors are primarily chemotherapeutic agents now being applied outside their designated field of use may also have been a contributing factor. On this latter point, it is a common misnomer to describe masitinib, and similar tyrosine kinase inhibitors, as a chemotherapeutic agent because - unlike cytotoxic chemotherapies that kill all dividing cells, including healthy cells - masitinib is a targeted therapy. Moreover, depending on which kinases are targeted, tyrosine kinase inhibitors are equally well suited for the treatment of nononcology diseases, as has been previously demonstrated for masitinib in inflammatory and autoimmune diseases with mast cell involvement, such as rheumatoid arthritis [16], asthma [17], mastocytosis [30], and atopic dermatitis [31], as well as experimental allergic encephalomyelitis, an animal model of brain inflammation.

**Table 4:** Comparison masitinib safety profile in nononcology phase 2 studies

	Phase 2 nononcology studies^a^			Alzheimer study	
	Controlled Masitinib (*n *= 79)	Placebo (*n *= 25)	Noncontrolled Masitinib (*n *= 137)	Masitinib (*n *= 26)	Placebo (*n *= 8)
At least one AE	73 (92%)	19 (76%)	122 (89%)	17 (65%)	3 (38%)
Serious AEs	22 (27%)	3 (12%)	35 (26%)	6 (23%)	1 (13%)
AE (withdrawal)^b^	25 (32%)	2 (8%)	44 (32%)	7 (27%)	0
Severe AE	35 (44%)	5 (20%)	49 (36%)	4 (15%)	1 (13%)
Dose reduction^c^	8 (10%)	0 (0%)	11 (8%)	4 (15%)	0

AE, adverse event. ^a^Excluding current Alzheimer study. ^b^AE leading to patient withdrawal from study. ^c^AE leading to dose reduction.

## Conclusions

Masitinib, an oral tyrosine kinase inhibitor with high activity against mast cells, administered as add-on therapy to standard care during 24 weeks showed promising signs of retarding the rate of cognitive decline of AD with an acceptable tolerance profile. Masitinib may therefore represent an innovative avenue of treatment in AD. Confirmatory phase 3 trials are justified to further investigate the long-term efficacy and safety of masitinib as an adjunct therapy with cholinesterase inhibitors and/or memantine for treatment of mild-to-moderate AD.

## Abbreviations

Aβ: amyloid beta; AD: Alzheimer's disease; ADAS-Cog: Alzheimer's Disease Assessment Scale - cognitive subscale; ADCS-ADL: Alzheimer's Disease Cooperative Study Activities of Daily Living Inventory; AE: adverse event; BBB: blood-brain barrier; CDR: Clinical Dementia Rating; CIBIC-plus: Clinician's Interview-Based Impression of Change-plus caregiver input; LOCF: last observation carried forward; MMSE: Mini-Mental State Examination; PDGFR: platelet-derived growth factor receptors.

## Competing interests

The present study was financially supported by AB Science. Masitinib is under clinical development by the study sponsor, AB Science (Paris, France). The sponsor was involved in the study design; data collection, analysis, and interpretation; and manuscript preparation and submission. AM is an employee and shareholder of the study sponsor. OH and PD are consultants and shareholders of the study sponsor. JB and LH-D have received honorarium from AB Science. No other conflicts of interest have been declared.

## Authors' contributions

FP, JB, HV, NS, SP, MV, CM, and JM participated in the study design, enrolled patients, collected data, and were involved in editing the manuscript. LH-D participated in the patient evaluation forms and editing of the manuscript. J-PK, PD, AM, and OH contributed to the study design and editing of the manuscript. All authors critically reviewed and approved the final manuscript.

## Supplementary Material

Additional file 1 **Alternative sensitivity analyses on ADAS-Cog according to the intent-to-treat population**. Tabulated data for ADAS-Cog response rate and ADAS-Cog change relative to baseline according to the sensitivity analysis approaches of: (1) the observed cases dataset; 2) last observation carried forward; and (3) considering missing data as nonresponders. Click here for file
