# Supplementary material for: Masitinib as an adjunct therapy for mild-to-moderate Alzheimer's disease: a randomised, placebo-controlled phase 2 trial
Source: Alzheimers Res Ther. 2011 Apr 19;3(2):16. doi: 10.1186/alzrt75 (PMC3226277; doi:10.1186/alzrt75)
Supplement: Additional file 1 — Alternative sensitivity analyses on ADAS-Cog according to the intent-to-treat population. Tabulated data for ADAS-Cog response rate and ADAS-Cog change relative to baseline according to the sensitivity analysis approaches of: (1) the observed cases dataset; 2) last observation carried forward; and (3) considering missing data as nonresponders. [file alzrt75-S1.PDF]

Additional File 1

François Piette et al. Masitinib as an adjunct therapy for mild-to-moderate Alzheimer's disease: a randomised, placebo-controlled phase 2 trial

Table S1. Alternative sensitivity analyses for primary efficacy outcome ADAS-Cog at weeks 12 and 24 in the intent-to-treat population. For comparison against Table 2 - Summary of efficacy outcomes at weeks 12 and 24.

**Sensitivity Analysis Method**	**Week 12**			**Week 24**		
	**Masitinib**	**Placebo**	***P*-value**	**Masitinib**	**Placebo**	***P*-value**
**Observed Cases**[a]						
Evaluable patients (*n*)	17	6		9	5	
Improvement (decrease ≥4)	7 (41%)	1 (17%)	0.369	3 (33%)	1 (20%)	1.0
Worsening (increase ≥4)	1 (6%)	3 (50%)	0.040	1 (11%)	3 (60%)	0.095
Mean absolute change ± SD[b]	-2.6 ± 3.6	4.2 ± 6.6	0.016	-0.7 ± 7.8	6.5 ± 8.6	0.182
Mean treatment effect[c]	**6.8**			**7.2**		
**Last Observation Carried Forward**						
Evaluable patients (*n*)	24	7		24	7	
Improvement (decrease ≥4)	9 (38%)	2 (29%)	1.0	8 (33%)	2 (29%)	1.0
Worsening (increase ≥4)	4 (17%)	3 (43%)	0.302	4 (17%)	3 (43%)	0.302
Mean absolute change ± SD	-1.0 ± 6.2	2.3 ± 7.9	0.247	-0.4 ± 7.2	3.6 ± 9.1	0.238
Mean treatment effect	**3.3**			**4.0**		
**Missing Data As Non Responders**[d]						
Evaluable patients (*n*)	26	8		26	8	
Improvement (decrease ≥4)	7 (27%)	1 (13%)	0.645	6 (23%)	1 (13%)	1.0
Worsening (increase ≥4)	10 (39%)	5 (63%)	0.417	11 (42%)	5 (63%)	0.429

[a]For the observed cases dataset no data imputation has been performed for the closed study centre. [b]Negative change reflects improvement. [c]Treatment effect is difference between treatment and placebo mean absolute change in ADAS-Cog score relative to baseline. Positive change reflects a positive treatment effect. [d]For the missing data considered as non responders dataset, week 12 data for closed study centre (*n*=8 patients) was imputed using last observation carried forward for week 24.
